# Prevalence of *Coxiella burnetii* in cattle at South Korean national breeding stock farms

**DOI:** 10.1371/journal.pone.0177478

**Published:** 2017-05-11

**Authors:** Min-Goo Seo, In-Ohk Ouh, Seung-Hun Lee, Jong Wan Kim, Man Hee Rhee, Oh-Deog Kwon, Tae-Hwan Kim, Dongmi Kwak

**Affiliations:** 1 College of Veterinary Medicine, Kyungpook National University, Daegu, South Korea; 2 Animal and Plant Quarantine Agency, Gimcheon, South Korea; 3 Cardiovascular Research Institute, Kyungpook National University, Daegu, South Korea; Washington State University, UNITED STATES

## Abstract

This is the first study to evaluate the serologic and molecular prevalence of *Coxiella burnetii* in cattle at national breeding stock farms in South Korea. These government farms have well-organized biosecurity and management systems to prevent livestock diseases. Of the 736 cattle in this study, 77 tested positive for antibodies against *C*. *burnetii* antigens (10.5%, 95% CI: 8.3–12.7) and 11 were positive for a *C*. *burnetti* infection on PCR analysis (1.5%, 95% CI: 0.6–2.4). Since the 16S rRNA sequences of *C*. *burnetii* from all 11 PCR-positive samples were identical, three representative samples (C-CN-3 from the southern region, C-JJ-9 from Jeju Island, and C-CB-37 from the central region) are described in this paper. These three sequences had 99.3–100% identity to those of *C*. *burnetii* deposited in GenBank. These sequences clustered with those from USA, Japan, and Greenland, underscoring the sequence similarity among *C*. *burnetii* isolates in these countries. Because *C*. *burnetii* was detected in cattle at well-managed national breeding stock farms, cattle at non-government operated farms may be more likely to be exposed to *C*. *burnetii* in South Korea. Thus, continuous surveillance and control strategies in animals and humans are required to prevent the transmission of *C*. *burnetii* to humans.

## Introduction

*Coxiella burnetii*, a zoonotic obligate intracellular bacterium, is the causative agent of Q fever in humans [1]. Q fever has been reported worldwide, except in New Zealand, and differences in host type and host factors affect the prevalence of disease in different regions and countries [1]. Reservoirs include ticks and domestic livestock, which are key sources of *C*. *burnetii* transmission [2]. Cattle infected with *C*. *burnetii* are usually asymptomatic; however, infection in dairy goats and sheep may result in abortion or stillbirth, often without preceding signs. *C*. *burnetii* infection can also cause mastitis, infertility, stillbirth, and reproductive disorders in animals [3], leading to economic losses.

The prevalence of *C*. *burnetii* infection in ruminants is of concern since Q fever is a zoonotic disease and ruminants are a reservoir for human infection [3]. The bacteria spread among animals by inhalation of infectious airborne dust or aerosol [4]. Infected animals shed *C*. *burnetii* in milk, feces, urine, semen, vaginal mucus, and birth products [3]. Therefore, *C*. *burnetii* infection in domestic animals is a public health concern. Dairy farmers, veterinarians, slaughterhouse workers, and anyone regularly interacting with animals or animal products are at risk for exposure to *C*. *burnetii* and development of Q fever [5]. Infected aerosols and ingestion of raw milk or dairy products are the primary routes of transmission from animals to humans [6].

National breeding stock farms in South Korea are operated by the government and are geographically isolated from nearby non-government operated farms. There are 17 national breeding stock farms throughout the country. These farms play an important role in improving and enhancing cattle productivity and developing breeding stock. These farms monitor disease and develop control measures to prevent the spread of disease. The national breeding stock farms have a well-organized biosecurity system. Previous studies of the seroprevalence of *C*. *burnetii* in cattle at non-government operated farms in Korea [7–9] did not describe the molecular detection of *C*. *burnetii*. Therefore, the purpose of this study was to assess the prevalence and genotypes of *C*. *burnetii* in cattle at national breeding stock farms in South Korea.

## Materials and methods

### Ethics statement

This study, conducted in 2014, did not receive approval from the Institutional Animal Care and Use Committee (IACUC) at Kyungpook National University (KNU), as the IACUC at KNU evaluates laboratory animals maintained in indoor facilities, and not outdoor animals. After receiving consent from the national breeding stock farms, blood samples were collected by practicing veterinarians during treatment or regular medical checkups. This study did not involve endangered or protected species.

### Sample size determination and sample collection

In 2014, the total number of cattle in South Korea was recorded as 3,189,951 [10]. The sample size was determined using the following formula, with an expected disease prevalence of 50%, an accepted absolute error of 5%, and a confidence level of 99% with a simple random sampling design [11]:

$$n=\frac{1.96^{2}p_{exp}(1-p_{exp})}{d^{2}}$$

where *n* = required sample size, *p*_exp_ = expected prevalence, and *d* = desired absolute precision.

**Fig 1:**
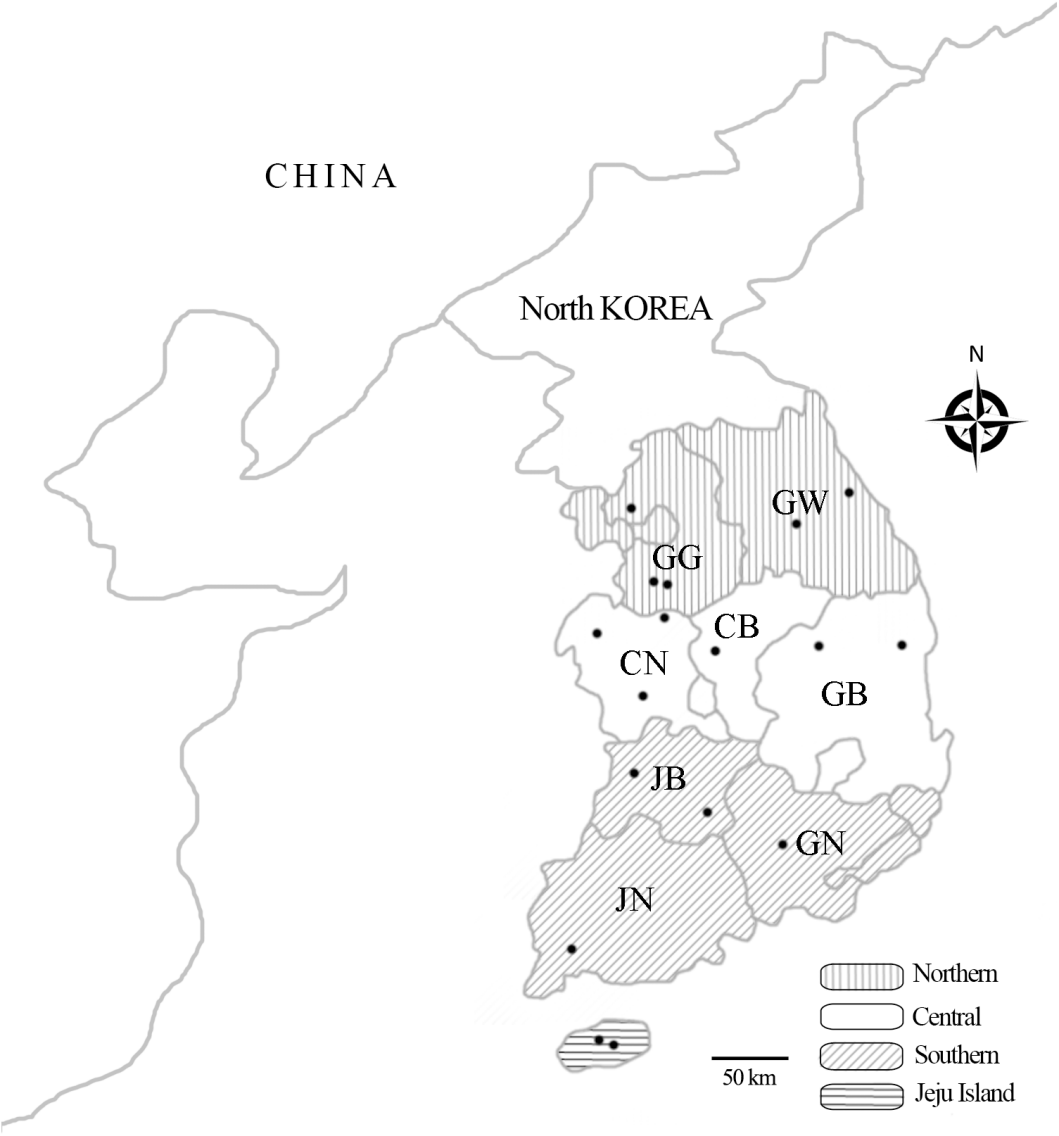
Geographic regions of the national breeding stock farms in South Korea. Blood samples were obtained from cattle at 17 national breeding stock farms from four different regions in Korea. The four regions were the following: Northern [Gyeonggi (GG) and Gangwon (GW)]; Central [Chungbuk (CB), Chungnam (CN), and Gyeongbuk (GB)]; Southern [Jeonbuk (JB), Jeonnam (JN), and Gyeongnam (GN)]; and Jeju Island. The location of the 17 national breeding stock farms are denoted by dots.

The calculated minimum sample size for this study was 663. Samples from 736 cattle were obtained in this study. Cattle were from 17 national breeding stock farms, from nine mainland provinces and Jeju Island, which is located in the southernmost region of South Korea (Fig 1). The farms were well managed in terms of biosecurity, disease, and breeding; however, there were geographic and climatic differences among farms. Blood was obtained from the jugular vein. Whole blood was used for PCR, and serum for serology. Samples were stored at -20°C until use. Age, sex, breed, and region were recorded for analysis.

### Serologic assay

Antibodies in serum against *C*. *burnetii* were detected using enzyme-linked immunosorbent assay (ELISA) using the ID Screen Q Fever Indirect Multi-species kit (IDvet, Montpellier, France), according to the manufacturer’s instructions. Sensitivity of this kit was 100% (30 bovine serum samples), and specificity was 100% (250 bovine serum and 88 caprine milk samples; IDvet, internal validation report). The ratio of the sample optical density (OD) to the positive control OD (S/P) was calculated for each sample as follows: Value (%) = (OD sample − OD negative control) / (OD positive control − OD negative control) × 100. Samples with S/P greater than 50% were considered positive; between 40% and 50%, doubtful; less than 40%, negative. Samples that were considered doubtful were treated as negative.

### Molecular assay

Genomic DNA was extracted from the whole blood using a commercial DNeasy Blood and Tissue kit (QIAGEN, Melbourne, Australia), according to the manufacturer’s instructions. The extracted DNA was stored at -20°C until use. The AccuPower HotStart PCR Premix kit (Bioneer, Daejeon, Korea) was used for PCR amplification. In nested PCR (nPCR), primer sets were used to amplify the 16S rRNA of the genus *Coxiella*, including *C*. *burnetii* and *Coxiella*-like bacteria (CLB) [12, 13]. First-round PCR was performed with the primers Cox16SF1 (5´-CGTAGGAATCTACCTTRTAGWGG-3´) and Cox16SR2 (5´-GCCTACCCGCTTCTGGTACAATT-3´), which produced amplicons of 1321–1429 bp. Then, nPCR was performed using the primers Cox16SF2 (5´-TGAGAACTAGCTGTTGGRRAGT-3´) and Cox16SR2, producing amplicons of 624–627 bp. Samples yielding amplicons of the expected size were bidirectionally sequenced using the primers Cox16SF1 and Cox16SR1 (5´-ACTYYCCAACAGCTAGTTCTCA-3´), which produced amplicons of 719–826 bp. PCR was performed using the Mastercycler Pro (Eppendorf, Hamburg, Germany) with predenaturation at 93°C for 3 min, followed by 30 cycles of denaturation at 93°C for 30 s, annealing at 56°C for 30 s, polymerization at 72°C for 1 min, and a final post-polymerization at 72°C for 5 min. After the second amplification, PCR products were separated on 1.5% agarose gels, stained with ethidium bromide, and visualized through UV transillumination.

### Sequencing and phylogenetic analysis

The purified PCR products were sequenced by Macrogen (Seoul, Korea) using the primers (Cox16SF1 and Cox16SR1) that were used in nPCR. The results were analyzed using the multiple sequence alignment programs, CLUSTAL Omega (ver. 1.2.1), and BioEdit (ver. 7.2.5). Phylogenetic analyses and homology comparisons were performed using MEGA (ver. 6.0) and the maximum-likelihood method. The stability of the phylogenetic tree was estimated using bootstrap analysis with 1,000 replicates.

### Statistical analysis

The chi-square test was used to analyze differences among the groups. A *p*-value of < 0.05 was considered statistically significant. The analytical software package GraphPad Prism version 5.04 (GraphPad Software Inc., La Jolla, CA, USA) was used for the statistical analysis. A 95% confidence interval (CI) was calculated for all estimates.

## Results

### Serologic and molecular assays

Of the 736 cattle in the study, 77 tested positive for *C*. *burnetii* antibodies (10.5%, 95% CI: 8.3–12.7) and 11 were positive by PCR (1.5%, 95% CI: 0.6–2.4) (Table 1). In addition, nine of 17 farms had *C*. *burnetii* antibody positive cattle (52.9%, 95% CI: 29.2–76.7) and five farms had cattle positive by PCR (29.4%, 95% CI: 7.8–51.1). The prevalence of ELISA (*p* < 0.0001) and PCR (*p* = 0.0015) positive cattle was significantly associated by herd status (S1 Table). Seroprevalence was significantly higher in cattle on Jeju Island (21.3%, 95% CI: 14.5–28.0) than in any of the other three geographical regions (*p* < 0.0001). Seroprevalence was significantly higher in black cattle (27.1%, 95% CI: 14.5–39.7) than in any other breed (*p* < 0.0001). Seropositivity for *C*. *burnetii* was significantly higher in female cattle (12.9%, 95% CI: 10.2–15.6) than in male cattle (1.3%, 95% CI: 0–3.1) (*p* < 0.0001) and significantly increased with age (*p* < 0.0001). Although the *C*. *burnetii* infection rate was low (1.5%; 11/736) when assessed by PCR, the trends with respect to geographical region, breed, sex, and age were similar (Table 1).

**Table 1:** Prevalence of *Coxiella burnetii* in 736 cattle at national breeding stock farms in South Korea during 2014.

Group		No. tested	No. of cattle ELISA-positive	95% CI^a^	*p*-value	No. of cattle PCR-positive	95% CI^a^	*p*-value
Region	Northern	180	8 (4.4)	1.4–7.5	< 0.0001	0	0	0.2041
	Central	250	22 (8.8)	5.3–12.3		4 (1.6)	0.1–3.2	
	Southern	165	17 (10.3)	5.7–14.9		3 (1.8)	0–3.9	
	Jeju Island	141	30 (21.3)	14.5–28.0		4 (2.8)	0.1–5.6	
Breed	Brown cattle	523	35 (6.7)	4.6–8.8	< 0.0001	6 (1.2)	0.2–2.1	0.1294
	Dairy cattle	155	27 (17.4)	11.5–23.4		3 (1.9)	0–4.1	
	Black cattle	48	13 (27.1)	14.5–39.7		1 (2.1)	0–6.1	
	Tiger cattle	10	2 (20.0)	0–44.8		1 (10.0)	0–28.6	
Sex	Female	581	75 (12.9)	10.2–15.6	< 0.0001	10 (1.7)	0.7–2.8	0.3266
	Male	155	2 (1.3)	0–3.1		1 (0.6)	0–1.9	
Age	<3	104	2 (1.9)	0–4.6	< 0.0001	0	0	0.2946
	3–5	355	24 (6.8)	4.2–9.4		5 (1.4)	0.2–2.6	
	5<	277	51 (18.4)	13.9–23.0		6 (2.2)	0.5–3.9	
Total		736	77 (10.5)	8.3–12.7		11 (1.5)	0.6–2.4	

^a^ CI = confidence interval.

### DNA sequencing and phylogenetic analysis

**Fig 2:**
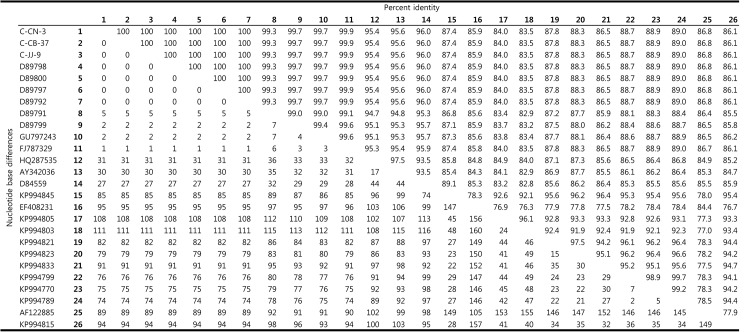
Comparison of the genus *Coxiella* 16S rRNA nucleotide sequences. Percent identities between sequences of *Coxiella burnetii* 16S rRNA gene fragment are shown in the upper matrix. The lower matrix shows the number of nucleotide base differences.

Results from PCR indicated that 11 animals were positive for *C*. *burnetii* infection. Because the sequences of these samples were identical, three samples were used as representative sequences for alignment and phylogenetic analysis. Fig 2 shows a comparative analysis of the nucleotide sequences for the 16S rRNA from the three samples (C-CN-3 from southern region, C-JJ-9 from Jeju Island, and C-CB-37 from central region) and from 23 bacterial species listed in GenBank. The three *C*. *burnetii* 16S rRNA sequences (accession nos. KT-835662, KU291428, and KU291429) showed 100% identity to each other and all were deposited in GenBank. The sequences also showed significant identity (99.3–100%) to eight additional *C*. *burnetii* isolates, which were also deposited in GenBank (Fig 2).

**Fig 3:**
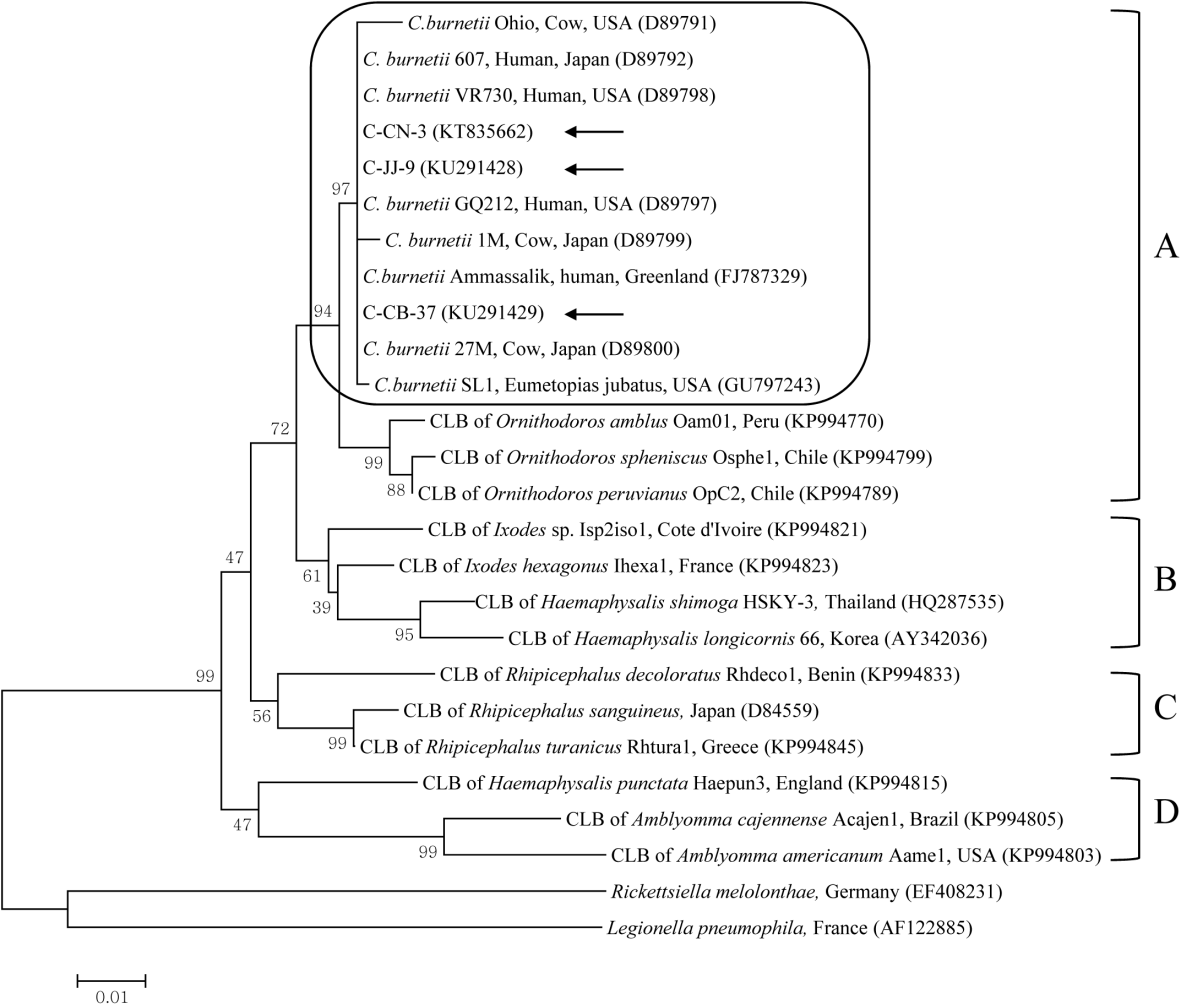
Phylogenetic tree constructed using the maximum-likelihood method based on the 16S rRNA sequences of the genus *Coxiella*. *Coxiella burnetii* sequences from this study are marked by arrows. *C*. *burnetii* species are grouped in a square. The four clades of the genus *Coxiella* are labeled A to D. Accession numbers of other sequences from GenBank are shown with the sequence names and countries. Numbers on the branches indicate bootstrap support (1,000 replicates). Scale bar is the phylogenetic distance in the sequence. CLB = *Coxiella*-like bacteria.

Phylogenetic analysis using the maximum-likelihood method indicated that the three isolates belonged to clade A clustered with isolates from USA (D89797, GU797243, D89798, and D89791), Greenland (FJ787329), and Japan (D89792, D89799, and D89800) (Fig 3).

## Discussion

The prevalence of *C*. *burnetii* infection in cattle at national breeding stock farms in South Korea was 10.5% (77/736) as detected by ELISA and 1.5% (11/736) detected through PCR analysis. The seroprevalence was significantly higher in cattle on Jeju Island than in other regions of South Korea. However, this is a lower overall seroprevalence than a previous study in native Korean cattle (18.9%; 40/212) at non-government operated farms on Jeju Island [9]. The wet marshy environment on Jeju Island is hospitable to ticks, and cattle are allowed to graze more freely than in other regions, thereby increasing the risk of tick exposure and *C*. *burnetii* infection.

Among native Korean cattle breeds, including brown cattle (Hanwoo), tiger cattle (brindle pattern, Chikso), and black cattle (Heugu), the black cattle had a significantly higher prevalence of *C*. *burnetii* infection than any other breed, including dairy cattle (Holstein). Black cattle, the traditional breed of Jeju Island, are only raised on Jeju Island. Differences in breed may influence the rate or prevalence of infection. Brown and dairy cattle are raised throughout Korea, including Jeju Island, but tiger cattle are raised in a restricted region of Jeonbuk, Korea. In our study, the prevalence in brown and dairy cattle was similar to the previous, ELISA-based studies of brown cattle (6.2%; 68/1,095) [9] and dairy cattle (24.2%; 119/492) [8].

Seroprevalence for *C*. *burnetii* was significantly higher in female cattle than in male cattle, and it increased with age. Hormonal differences may affect susceptibility to infection. There is a difference in clinical expression of Q fever between men and women. Although men and women are equally exposed to *C*. *burnetii*, as evidenced by similar seroprevalence [14], Q fever symptoms are more common in men. Occupational or environmental influences, breeding, and age may play a role in the development of infection in many species, including cattle and humans. The difference in age may relate to early exposure and continuous infection. The risk of infection coincides with the time the host reaches adulthood [15], and adult cattle are exposed to *C*. *burnetii* and tick bites for extended periods [16]. Similarly, the prevalence of *C*. *burnetii* in native Korean goats also increased with age in South Korea [17].

Three complementary lines of evidence suggest a substantially longer phylogenetic history between the tick and *Coxiella* than between vertebrate and *Coxiella* [13]. First, there is wide genetic variation in tick-borne *Coxiella* strains, or CLB, compared to *C*. *burnetii* strains, including the subdivision of *Coxiella* into four greatly divergent clades (A to D). Second, *Rickettsia* and *Coxiella* bacteria are widely distributed in tick species, genera, and families. Third, the entire cluster of *C*. *burnetii* strains belongs to clade A (one of the clades of CLB), which suggests that the progenitor of *C*. *burnetii* was a tick-related bacterium that successfully infected vertebrate cells [13]. Generally, detection of CLB has been restricted to ticks, and CLB may pose a lesser threat to vertebrates than *C*. *burnetii* [18]. However, potential tick-to-vertebrate transmission of CLB is likely because ticks occur worldwide and feed on various hosts [19]. CLB are proposed as the progenitor of *C*. *burnetii* [13]. A recent study using *Coxiella* 16S rRNA sequencing identified the first instance of CLB in horses in South Korea, with a prevalence of 0.7% (6/816) [20]. In that study, CLB were detected in a vertebrate for the first time with the same primers used in this study.

The genus *Coxiella* is extremely diverse and widespread. While CLB share genetic features with *C*. *burnetii*, the sequences in common are variable [19]. While we tried to amplify several genes of *C*. *burnetii*, including *IS1111* (transposase insertion element), *com1* (encoding a 27 kDa outer membrane protein), *icd* (isocitrate dehydrogenase), and *sod* (encoding for superoxide dismutase), these specific genes were not amplified by PCR (data not shown). In addition, CLB have been characterized solely by their 16S rRNA gene sequences [19]. Although other genes may discriminate between *C*. *burnetii* and CLB, they are currently differentiated on the basis of phylogenetic analysis, considering the 16S rRNA [19]. In this study in cattle, we determined the prevalence of *Coxiella*, including *C*. *burnetii* and CLB, by targeting the 16S rRNA. Phylogenetic analysis indicated that all 11 sequences of *C*. *burnetii* detected from cattle were closely related to sequences of several *C*. *burnetii* in the GenBank, which belong to clade A. These sequences clustered with *C*. *burnetii* isolates from USA, Japan, and Greenland, suggesting a near epidemiologic connection among these isolates.

This is the first nationwide, large-scale study on the serologic and molecular detection of *C*. *burnetii* infection among cattle at national breeding stock farms in South Korea. Because *C*. *burnetii* was detected at national breeding stock farms that have heightened biosecurity and geographical isolation, cattle at non-government operated farms may be more likely to be exposed to *C*. *burnetii*. Recently, ticks infesting horses have been shown to carry CLB (52.4%, 121/213) in South Korea with the same primers used in the present study [21], suggesting that ticks might be a reservoir for transmitting *Coxiella* spp. In addition, since *C*. *burnetii* was detected in pigs by serology (6.8%, 70/1,030) and 16S rRNA sequencing (0.3%, 3/1,124) [22], potential transmission of *C*. *burnetii* from pigs to humans cannot be excluded. It is difficult to minimize *C*. *burnetii* exposure in farm-raised animals, as there are no clear clinical signs of infection, and farmers may not recognize Q fever or its economic impact. Thus, continuous monitoring and control strategies in other mammals and ticks are required to prevent the transmission of *C*. *burnetii*, the causative agent of Q fever, to humans.

## Conclusions

The present study is the first serologic and molecular assessment of *C*. *burnetii* in cattle at national breeding stock farms, where the South Korean government provides well-organized biosecurity for livestock diseases. Since *C*. *burnetii* was detected at national breeding stock farms that had high-quality biosecurity, cattle at non-government operated farms may be more likely to be exposed to *C*. *burnetii*. Infection with *C*. *burnetii* may be misdiagnosed in both humans and animals. Surveillance systems are essential to assess the actual incidence of Q fever.

## Supporting information

S1 Table Additional descriptive information of national breeding stock farms in South Korea. (DOCX)
