# Supplementary material for: Prevalence of Coxiella burnetii in cattle at South Korean national breeding stock farms
Source: PLoS One. 2017 May 11;12(5):e0177478. doi: 10.1371/journal.pone.0177478 (PMC5426765; doi:10.1371/journal.pone.0177478)
Supplement: S1 Table — (DOCX) [file pone.0177478.s001.docx]

**S1 Table. Additional descriptive information of national breeding stock farms in South Korea.**

| Region | Province | Farm no. | Herd size | No. tested | Breed | Mean age | Age range | No. of cattle ELISA-positive | No. of cattle PCR-positive |
| --- | --- | --- | --- | --- | --- | --- | --- | --- | --- |
| Northern |  |  | 191^a^ |  |  | 5 | 0–15^b^ |  |  |
|  | Gyeonggi | 1 | 191 | 30 | Brown cattle | 7 | 4–11 | 0 | 0 |
|  |  | 2 | 63 | 45 | Dairy cattle | 3 | 0–9^b^ | 1 | 0 |
|  |  | 3 | 151 | 45 | Brown and dairy cattle | 7 | 4–15 | 7 | 0 |
|  | Gangwon | 4 | 485 | 30 | Brown cattle | 6 | 2–14 | 0 | 0 |
|  |  | 5 | 870 | 30 | Brown cattle | 6 | 2–9 | 0 | 0 |
| Central |  |  | 327.5^a^ |  |  | 5 | 0–17^b^ |  |  |
|  | Chungbuk | 6 | 280 | 45 | Brown cattle | 7 | 1–17 | 3 | 1 |
|  | Chungnam | 7 | 235 | 30 | Brown cattle | 4 | 3–5 | 0 | 0 |
|  |  | 8 | 2,866 | 45 | Brown cattle | 2 | 1–4 | 0 | 0 |
|  |  | 9 | 375 | 45 | Dairy cattle | 6 | 4–10 | 18 | 2 |
|  | Gyeongbuk | 10 | 502 | 40 | Brown cattle | 3 | 0–10^b^ | 0 | 0 |
|  |  | 11 | 129 | 45 | Dairy cattle | 4 | 3–6 | 1 | 1 |
| Southern |  |  | 200^a^ |  |  | 5 | 1–14 |  |  |
|  | Jeonbuk | 12 | 247 | 45 | Tiger and Brown cattle | 5 | 1–10 | 15 | 5 |
|  |  | 13 | 304 | 30 | Brown cattle | 8 | 4–14 | 0 | 0 |
|  | Jeonnam | 14 | 153 | 45 | Brown cattle | 5 | 1–11 | 1 | 0 |
|  | Gyeongnam | 15 | 130 | 45 | Brown cattle | 3 | 1–5 | 1 | 0 |
| Jeju Island |  |  | 249.5^a^ |  |  | 7 | 3–20 |  |  |
|  | Jeju Island | 16 | 340 | 65 | Brown and Black cattle | 7 | 3–12 | 30 | 2 |
|  |  | 17 | 159 | 76 | Brown and Black cattle | 7 | 3–20 | 0 | 0 |
| *p*-value |  |  |  |  |  |  |  | < 0.0001 | 0.0015 |

^a^Median herd size of each region; ^b^0 = less than 1 year old
